# Brain structural plasticity in large-brained mammals: Not only narrowing roads

**DOI:** 10.4103/NRR.NRR-D-24-01438

**Published:** 2025-03-25

**Authors:** Marco Ghibaudi, Alessandro Zanone, Luca Bonfanti

**Affiliations:** 1 Neuroscience Institute Cavalieri Ottolenghi (NICO), Orbassano, Italy; 2 Department of Veterinary Sciences, University of Turin, Torino, Italy

**Keywords:** adult neurogenesis, amygdala, brain plasticity, cerebral cortex, comparative approach, evolution, immature neurons

## Abstract

The capacity of the central nervous system for structural plasticity and regeneration is commonly believed to show a decreasing progression from “small and simple” brains to the larger, more complex brains of mammals. However, recent findings revealed that some forms of neural plasticity can show a reverse trend. Although plasticity is a well-preserved, transversal feature across the animal world, a variety of cell populations and mechanisms seem to have evolved to enable structural modifications to take place in widely different brains, likely as adaptations to selective pressures. Increasing evidence now indicates that a trade-off has occurred between regenerative (mostly stem cell–driven) plasticity and developmental (mostly juvenile) remodeling, with the latter primarily aimed not at brain repair but rather at “sculpting” the neural circuits based on experience. In particular, an evolutionary trade-off has occurred between neurogenic processes intended to support the possibility of recruiting new neurons throughout life and the different ways of obtaining new neurons, and between the different brain locations in which plasticity occurs. This review first briefly surveys the different types of plasticity and the complexity of their possible outcomes and then focuses on recent findings showing that the mammalian brain has a stem cell–independent integration of new neurons into pre-existing (mature) neural circuits. This process is still largely unknown but involves neuronal cells that have been blocked in arrested maturation since their embryonic origin (also termed “immature” or “dormant” neurons). These cells can then restart maturation throughout the animal’s lifespan to become functional neurons in brain regions, such as the cerebral cortex and amygdala, that are relevant to high-order cognition and emotions. Unlike stem cell–driven postnatal/adult neurogenesis, which significantly decreases from small-brained, short-living species to large-brained ones, immature neurons are particularly abundant in large-brained, long-living mammals, including humans. The immature neural cell populations hosted in these complex brains are an interesting example of an “enlarged road” in the phylogenetic trend of plastic potential decreases commonly observed in the animal world. The topic of dormant neurons that covary with brain size and gyrencephaly represents a prospective turning point in the field of neuroplasticity, with important translational outcomes. These cells can represent a reservoir of undifferentiated neurons, potentially granting plasticity within the high-order circuits subserving the most sophisticated cognitive skills that are important in the growing brains of young, healthy individuals and are frequently affected by debilitating neurodevelopmental and degenerative disorders.

## Introduction

A common belief in the neurosciences considers the ability of the brain to undergo structural plasticity changes (physical changes in the shape, number, and contacts of neurons and glial cells) and regeneration (restoration of the interrupted continuity of a missing organ mass) to show a progressive decrease, being higher in animal species endowed with small and “simple” nervous systems than in those possessing large and “complex” ones (Lindsey and Tropepe, 2006; Krubitzer, 2009; Tanaka and Ferretti, 2009; Bonfanti, 2011; Barbosa-Sabanero et al., 2012; Alunni and Bally-Cuif, 2016; Paredes et al., 2016; Blackshaw, 2022). Of course, brain complexity can be considered a relative, rather than an absolute, concept (Bullock, 2007; Krubitzer, 2009) that does not always show a direct correlation with the level of plasticity. For instance, stem cell–driven nerve cell renewal is present in virtually all animals, despite the structural complexity of the mammalian olfactory bulb and hippocampus (Amrein et al., 2011; Barker et al., 2011; Aimone et al., 2014; Lledo and Valley, 2016). Nevertheless, a progressive phylogenetic decrease in the capacity for plasticity (mostly regenerative) is noticeable in mammalian brains due to selective pressures and neuroanatomical constraints. For instance, the capacity for stem cell–driven nerve cell renewal is highly reduced in the large-brained humans (Sanai et al., 2011; Cipriani et al., 2018; Sorrells et al., 2018) and even absent in dolphins (Patzke et al., 2015; Parolisi et al., 2017). A previous analysis that considered brain regeneration in the animal world defined the survey conducted across different species, orders, and classes as “a long trip through narrowing roads” to underline the existence of a progressive restriction in this potential (Bonfanti, 2011). Some invertebrates, such as cnidarians, echinoderms, and some worms, are well known to regenerate their whole bodies when cut in half (e.g., hydra and planaria; Benfey, 1999; Birnbaum and Sanchez Alvarado, 2008) or can regenerate entire parts, such as an arm (e.g., urodele amphibians; Nye et al., 2003; Kragl et al., 2009), whereas these processes are not possible in mammals. Similarly, some complex vertebrates (e.g., fish) have lost these most striking regenerative capacities but still retain the ability to continuously renew a large variety of neural elements through types of brain regeneration that are not possible in mammals (Becker and Becker, 2008; Kaslin et al., 2008; Lindsey et al., 2018; Lange and Brand 2020). Furthermore, in mammals, nerve cell renewal (so-called adult neurogenesis; Lois and Alvarez-Buylla, 1994) is restricted to two or three small neurogenic sites whose activity progressively decreases with age (Ben Abdallah et al., 2010; Encinas et al., 2011; Lee and Blackshaw, 2012; Bond et al., 2015; Lim and Alvarez-Buylla, 2016), indicating that real brain regenerative potential is essentially absent (Masaki and Ide 2007; Weil et al., 2008; Bonfanti, 2011).

A deeper comparison of neuroplasticity among mammals reveals that the relationship between brain anatomy/size and structural plasticity is not always linear. Apart from synaptic plasticity—which is considered ubiquitous in the nervous system (DeFelipe et al., 2002; Bufill et al., 2011; Sherwood et al., 2020) and thus has the potential to increase with the rising numbers of neuronal contacts in larger brains (see below)—other important interspecies differences are known to exist regarding the occurrence and rate of adult neurogenesis (Lindsey and Tropepe, 2006; Barker et al., 2011; Amrein, 2015; Bonfanti et al., 2024). Some species (e.g., bats and dolphins; Amrein et al., 2007, 2011; Patzke et al., 2015; Parolisi et al., 2017) have very low levels of postnatal neurogenesis, whereas other species (e.g., some primates and humans; Knoth et al., 2010; Sanai et al., 2011; Cipriani et al., 2018; Sorrells et al., 2018; Hao et al., 2022) show this postnatal capability only in the young life stages. In particular, in long-living species, these robust neurogenic processes are found mostly in the juvenile stages (see Snyder, 2019; Charvet and Finlay, 2018; Bond et al., 2022; and below).

Regardless of whether the phylogenetic reduction of stem cell–driven neurogenesis is evident from fish to mammals and from mice to humans (reviewed in Lindsey and Tropepe, 2006; Bonfanti, 2011; Bonfanti et al., 2024), the finding that a novel form of “neurogenesis without division” (the formation of “immature” neurons; Bonfanti and Nacher, 2012; Bonfanti and Seki, 2021; La Rosa and Bonfanti, 2021; Benedetti and Couillard-Despres, 2022) is particularly abundant in large-brained mammals challenges the current consensus regarding the phylogenetic trends of brain structural plasticity (see below, the section on protracted neurogenesis). The complex, non-linear interactions between different nervous systems may create challenges in understanding the logic followed by structural plasticity through its evolution; however, this complexity and lack of knowledge emphasize the need for more studies that compare widely different species and animal models (Preuss, 2004; Lindsey and Tropepe, 2006; Smulders, 2009; Brenowitz and Zakon, 2015; Faykoo-Martinez et al., 2017; La Rosa and Bonfanti, 2018; Bonfanti and Charvet, 2021). This concept has been repeatedly highlighted in elegant and farsighted commentaries, as shown by the following representative statements:

“Because every species’ brain is unique, with its specific adaptations, it is especially important to be aware of brain evolution when using non-human animals to understand the human brain” (Smulders, 2009).

“Most biomedical researchers stress similarities to the point of forgetting that there is another side to the evolutionary coin: the differences that make each species unique” (Preuss, 2004).

“The coalescence on a small set of model species comes with several costs that are often not considered, especially in the current drive to use mice explicitly as models for human diseases. Comparative studies of strategically chosen non-model species can complement model species research and yield more rigorous studies” (Brenowitz and Zakon, 2015).

**Figure 1:**
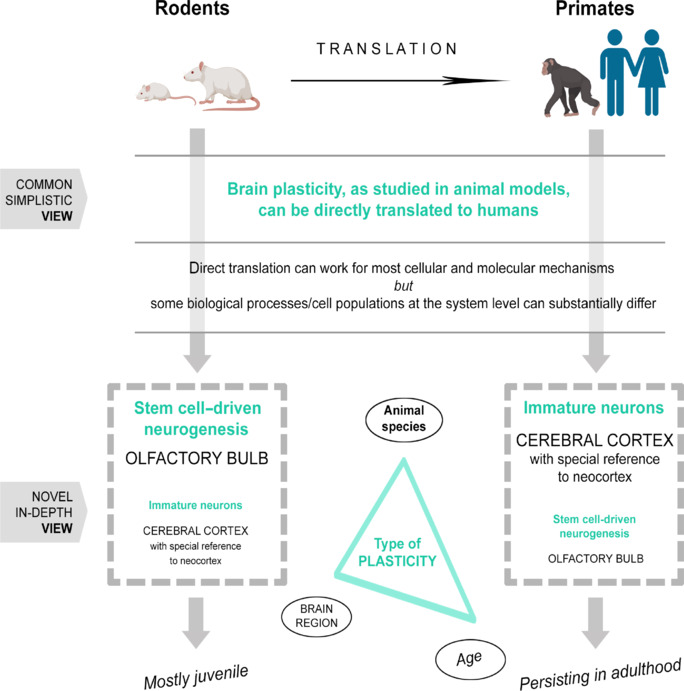
Three main elements that are important in brain structural plasticity interspecies variation and potentially affect translation. Interpretation of results obtained in experimental research carried out on laboratory rodents must consider interspecies differences. Besides the many similarities shared by mammals (most molecular and cellular mechanisms), some aspects of their biology (e.g., brain plasticity) can differ markedly, thus hampering the correct translation to humans. Many scientists still believe that knowledge produced in animal models (mostly rodents) can be directly transferred through clinical trials in humans. Nevertheless, some emerging differences in brain anatomy and functioning, specifically concerning the types, amounts, and persistence of brain structural plasticity, suggest that this is not always the case. The discovery that stem cell–driven neurogenesis and non-dividing “immature” neurons are differentially distributed (spatially and temporally) across mammals (here represented by the size of characters) indicates that the brain regions displaying the substrate for plasticity (neurogenic niches versus cerebral cortex, and likely amygdala; see Figure 3 for more detail), are important elements at the base of interspecies differences. Created with BioRender.com.

This issue enables the opportunity to emphasize aspects that are often skipped, underestimated, or misunderstood in the field of neurodevelopmental biology. Foremost is the fact that remarkable interspecies variations in brain plasticity are apparent when comparing mice and humans, and this impacts the correct translation of the results derived from research performed in laboratory rodents (Bolker, 2017; Faykoo-Martinez et al., 2017; La Rosa and Bonfanti, 2018; **Figure 1**). The aim of the present review is to address some elements thought to have played an evolutionary role in determining the choice between different types of plasticity in different species and brain regions by highlighting a recent twist involving a “trend reversal in the narrowing roads.”

## Search Strategy

The studies cited in this review were mainly published between 2010 and 2024 and were retrieved using a search of the PubMed database. The present article deals with some topics that have appeared in a large number of published reports in the last decades (e.g., brain plasticity: 85,000 papers stored in PubMed; adult neurogenesis: 13,000; brain regeneration: 20,000). Among these articles, only the most relevant and those dealing with comparative studies or evolutionary aspects were selected. A certain number of reports published before 2010 also required citation since some information relevant to comparative studies has been produced in the past. Conversely, a substantial part of this review concerns the novel field of investigation involving “immature” or “dormant” neurons, and most reports on this topic have been published between 2018 and 2024.

## Brain Structural Plasticity and Regeneration in the Animal World: Mixed Reasons for the “Narrowing Roads”

Although the present review focuses predominantly on mammals, we found that extending our analysis to other vertebrates, including those capable of substantial brain regeneration, such as teleost fish, reptiles, axolotls, and newts (Grandel et al., 2006; Becker and Becker, 2008; Kaslin et al., 2008; Tanaka and Ferretti, 2009; Zupanc and Sîrbulescu, 2011; Joven and Simon, 2018), was useful for understanding the reasons for the phylogenetic reduction of plastic and regenerative potential during evolution. The prominent requirements for efficient regenerative capabilities remain the occurrence, amount, distribution, and activity of neural stem cells or various types of stem-like cells. Some fish species display multiple neurogenic sites endowed with active stem cells in wide areas of the brain and spinal cord (Lange and Brand, 2020; Vandestadt et al., 2021), and these stem cell reservoirs enable continuous neuronal cell renewal and regeneration throughout the organism’s life (Grandel et al., 2006; Zupanc and Sîrbulescu, 2011; Lange and Brand, 2020; Vandestadt et al., 2021). The fact that stem cells, at least in some animal species, can be a source for regeneration, along with the discovery that some stem cell niches can be present in the mammalian brain, has suggested that endogenous neural regeneration might be inducible in humans (Lindvall and Kokaia, 2010; Bao and Song, 2018). Nevertheless, this suggestion has proved to be somewhat misleading due to the discovery that mammalian stem cell–driven neurogenesis is spatially and temporally restricted and produces only a narrow range of neuronal types destined to serve specific neural networks (Obernier et al., 2014; Bond et al., 2015; Lim and Alvarez-Buylla, 2016). Another important consideration is that mammalian adult neurogenesis differs from other adult stem cell–supported processes in the body. In contrast to blood, epidermis, and the epithelia of the small intestine, which are characterized by a steady renewal of all cells throughout the lifespan (Potten and Loeffler, 1990), brain stem cells undergo a progressive depletion over time due to decreased numbers or entry into a quiescent state, which causes an exponential decline in the rate of neurogenesis during adult life (this occurs to a lesser extent in mice but is more evident in humans; Ben Abdallah et al., 2010; Encinas et al., 2011; Sanai et al., 2011; Sorrells et al., 2018; Zhou et al., 2022; Blasco-Chamarro and Fariñas, 2023).

**Figure 2:**
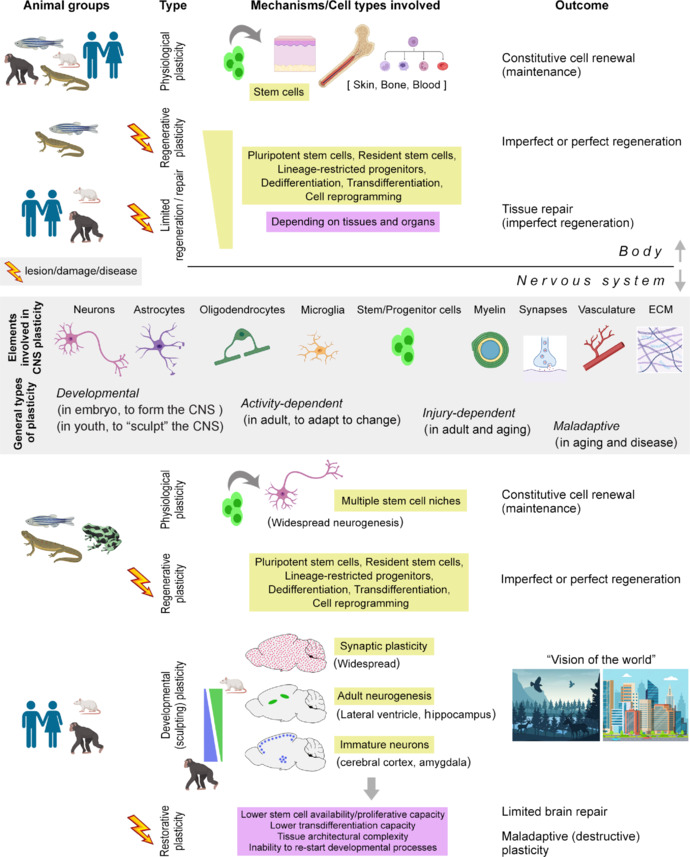
Different roles and outcomes for brain structural plasticity in the animal world, with special reference to mammals. Across phylogeny, different forms of structural plasticity have adapted to different tissues/organs in different species. The terminology and classification of the resulting multi-level (often overlapping) processes have been different through time and among authors, and the aim here is to give a general summary of such complexity. Plasticity can be linked to the physiological maintenance of healthy tissues (through constitutive cell renewal), as well as to a response to damage, leading to perfect regeneration, imperfect regeneration, repair, or even maladaptive plasticity (e.g., in the nervous system), depending on the species, tissue/organ, and cell types/biological processes involved. The lower part of the figure focuses on peculiar features of the nervous system, which, in mammals, has become a mostly non-renewable tissue that differs from most organs in the body. Overall, the continuous tissue maintenance through cell renewal and the regenerative capacity in the nervous system are highly reduced through phylogeny, while some forms of physiological “sculpting” plasticity are increased and become useful during youth and more evident in large-brained, long-living species. In sculpting plasticity, although the iconic brain is always that of rodents, the different importance of stem cell–driven neurogenic processes (green) and non-dividing neurons (blue) in different mammalian species (from mouse to chimpanzee) is represented on the left. Yellow background: cells and processes granting plasticity; purple background: limits/biological processes hampering brain repair. Created with BioRender.com.

In addition to the general trend observed from fish to humans described above, recent studies reveal a far more complex situation throughout the phylogenetic tree (Brockes and Kumar, 2008). In many tissues and organisms, the endogenous (stem cell–driven) cell renewal activity seems related to their regenerative ability; however, this is not a general rule, and the existence of neural stem cells is not enough to support brain regeneration. In brief, for regeneration, different organisms and organs employ a variety of cell types and biological strategies, including pluripotent stem cells, resident (tissue-specific) stem cells, lineage-restricted progenitors, processes of dedifferentiation and transdifferentiation (i.e., cells that can dedifferentiate and subsequently redifferentiate without cell division or via a progenitor cell produced by dedifferentiation), and cell reprogramming induced by experimental approaches (Jopling et al., 2011; Tanaka and Reddien, 2011; **Figure 2**). Very simple organisms, such as planarians, undergo regeneration using adult pluripotent stem cells, whereas other vertebrates utilize a variety of lineage-restricted progenitors (Mchedlishvili et al., 2007). For example, the extensive regeneration capacity of the salamander’s nervous system is linked to the persistent presence of ventricular ependymoglial cells (radial glia-like elements with stem cell–like features) throughout its entire lifespan (Joven and Simon, 2018). Even the outcome of the regenerative process can be heterogeneous, as axolotls can regenerate most brain cell populations, but the newborn neurons form an altered tissue architecture and fail to reestablish the circuit physiology present before injury (Amamoto et al., 2016).

The different capacities for regeneration observed in the phylogenetic tree are also correlated with specializations of the immune system. Immune cells generally have a negative effect on wound healing, since they determine the tissue response to injury, including the degree of inflammation and extent of scarring (Harty et al., 2003; Mescher and Neff, 2006; Blackshaw, 2022). Accordingly, regeneration of the nervous system differs in urodele and anuran amphibians, being possible during adulthood in the former but restricted to the larval stage in the latter. This loss of regenerative capacity is viewed as a turning point in evolutionary history, linked to the fact that immune reactivity is greater in anurans than in urodeles (Harty et al., 2003; Mescher and Neff, 2006). One recent theory in brain regeneration suggests a role for immunity, with the hypothesis that a selection occurs for greater resistance to the spread of central nervous system infections, leading to enhanced reactive gliosis and loss of injury-induced regeneration (Harty et al., 2003; Blackshaw, 2022). Importantly, reaccess to embryonic developmental programs is known to occur during the regenerative processes of salamanders and axolotls (Mchedlishvili et al., 2007; Tanaka and Ferretti, 2009), whereas, in mammals and birds, some domains become refractory to signals very early, which could further explain the loss of regenerative capacity. Finally, in addition to being influenced by the availability, amount, location, and properties of the stem cell pool, the potential for structural plasticity is also highly defined by the tissue environment, including extracellular matrix components, myelin-associated molecules, glial cell types, and the glial scars that frequently form after brain injury (Adams and Gallo, 2018; Cope and Gould, 2019; Falcone et al., 2019).

Hence, aspects beyond the availability and type of stem cells appear responsible for the “narrowing road” observed in the nervous system regenerative capacity in mammals. In brief, these aspects include the size and anatomical complexity of the brain, the topographical restriction of germinal layer–derived stem cell niches, the emergence of specialized glial cell populations versus immature radial/ependymal cells, the inhibitory factors existing in intact nervous tissue, and the strength of immune surveillance and the consequent tissue reactions, most of which are detrimental (e.g., reactive gliosis and inflammation). Multiple candidate sources of new cells could, in principle, act in concert to allow regeneration of a complex tissue (Tanaka and Reddien, 2011); however, in the mammalian brain, the hampering factor is a combination of a lack of stem cell availability, poor proliferative capacity (or transdifferentiation capacity), and tissue architectural complexity, with this whole interplay preventing the re-starting of developmental processes.

## Developmental Plasticity *Versus* Brain Regeneration and Repair

The word “plasticity” in the context of the nervous system (or its variant “structural plasticity,” with a distinction between structural and functional plasticity being highly semantical; Boonstra and Slagter, 2019; Axelrod et al., 2023) is often used for a very wide range of processes, spanning from brain postnatal development and maintenance to reactions at various lesion and disease states (Møller, 2008; Jopling et al., 2011; Fox and Stryker, 2017; Seblani et al., 2024; **Figure 2**). Nevertheless, the existence of plastic potential does not always result in a reparative outcome. In most cases, physiological plasticity (maintenance, hereafter referred to as plasticity) and regenerative/reparative plasticity (hereafter referred to as regeneration) are two distinct processes that require different conditions and that may or may not coexist. While plasticity in simple organisms can be the source of regeneration/repair, in mammals, it plays a prominent role in postnatal brain development and neural circuit refinement but is seldom useful in reparative contexts (and is thus referred to as restorative plasticity; **Figure 2**; see below). A shift between these two roles has occurred in phylogeny, and the explanation (or hypothesis) for the regenerative/reparative restriction was well conveyed by Weil et al. (2008); they stated that “vertebrate evolution has favored a strategy of protecting the central nervous system from potential injuries rather than evolving mechanisms to regenerate damaged tissue” and “the injured nervous system does not favor a strategy of regeneration, but rather one of minimizing further damage.”

A famous experiment that used transgenic mice to ablate proliferating astrocytes and prevent the formation of the glial scar in the spinal cord revealed that mechanical lesions that usually produce small, circumscribed lesions instead induced dramatically exacerbated inflammatory responses, blood–brain barrier disruption, demyelination, and cell loss in the transgenic mice (Faulkner et al., 2004). In general, a series of repair attempts and undue reactions directed to circumscribe damage and avoid the spread of inflammation or infections can lead to maladaptive plasticity (also termed destructive, harmful, negative, abnormal, aberrant, dysfunctional, or bad plasticity; Pascual-Leone et al., 2005; Møller, 2008; Tomaszczyk et al., 2014; Seblani et al., 2024). This concept, originally introduced by Catherine Malabou in the context of the limit between neurobiology and psychoanalysis (Malabou and Miller, 2012), warns about using the word plasticity only to describe “constructive and reparative” mechanisms, thereby underestimating its negative consequences in various neurological disorders (Seblani et al., 2024). In other words, plasticity, by increasing susceptibility to the environment, can have either beneficial or detrimental effects (Belsky et al., 2009). Regardless of whether the remodeling of neuronal networks can compensate for the loss of brain function occurring with aging, these plastic changes clearly can sometimes be detrimental. For instance, excessive/aberrant plasticity has been suggested as one possible cause of Alzheimer’s disease (Kawabata, 2022), while low-grade inflammation in multiple sclerosis drives mature astroglia to undergo adaptive cellular reprogramming into senescent, neurotoxic, anti-regenerative elements that further exacerbate neuronal damage and accelerate disease progression (Park et al., 2024). Unfortunately, the dual nature of plastic mechanisms creates difficulty in discerning the initial causes of the disease/lesion from the plastic reactions, which can subsequently be detrimental or beneficial. For this reason, the border between restorative and maladaptive plasticity is not always sharply defined.

**Figure 3:**
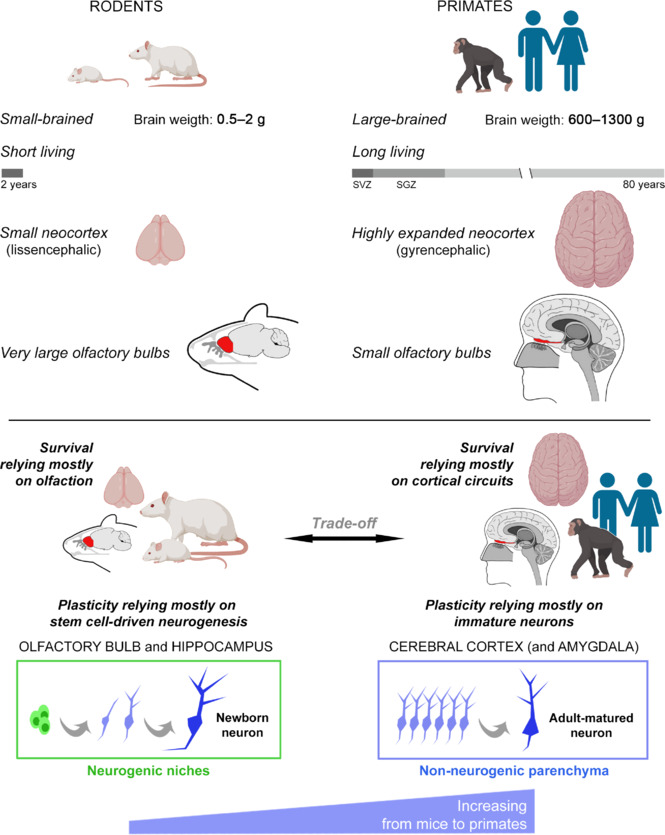
Some aspects considered to have affected evolutionary choices in brain plasticity between widely different mammals. Laboratory rodents and primates are represented here as extreme examples displaying marked differences in types and amounts of neurogenic plasticity because rodents are considered the primary animal models for neuroscience studies, while humans are the targets of translation. Top: Brain size, neocortical expansion, olfaction, and lifespan can be remarkably different in mice and primates, and these aspects have been linked to different choices that occurred during evolution concerning the type and location of brain plasticity (see text). The dark gray segment in the lifespan bar indicates the persistence of stem cell–driven adult neurogenesis; SVZ: subventricular zone; SGZ: subgranular zone. Bottom: according to an emerging view (Aboitiz and Montiel, 2015; Bonfanti et al., 2024), the relative importance of olfaction versus other functions linked to neocortical expansion for spatial navigation and survival has changed through evolution in rodents and primates, in parallel with a different prevalence of stem cell–driven neurogenesis (linked to olfactory bulb and hippocampus; green) or prenatally generated “immature” neurons (linked to cerebral cortex and amygdala; light blue). For this reason, the cortical immature neurons might represent an increasing substrate for structural plasticity ranging from small-brained to large-brained mammals. Created with BioRender.com.

The lesson drawn from comparative studies across phylogeny seems to be that postnatal developmental changes (so-called developmental or activity-dependent plasticity) were the primary reason for the development of neural plasticity in organisms, whereas brain repair (injury-dependent plasticity) might have arisen as a byproduct of evolution and therefore encountered more difficulties with increasing brain complexity. According to this view, developmental plasticity became less involved in repair in mammals and instead maintained its mission of refining neural circuits during juvenile stages based on experience, followed by a reduction of plasticity around adolescence to allow a “stabilization” useful for navigation of the (now) adult individual for the rest of its lifespan. This is another simple reason for the general reduction of regeneration capabilities in large, complex brains: they require certain degrees of stability throughout adult life, as this can be useful for taking advantage of cumulative experiences (this differs from the continuous somatic growth that characterizes some fish species and extends over longer times concerning rodents). This aspect may also fit with a kind of plasticity (e.g., non-dividing dormant neurons) found in higher-order brain regions and linked to social cognition, planning, decision-making, and personality (neocortex and amygdala), rather than the stem cell–driven neurogenic processes associated with instinctive behaviors (e.g., most functions linked to olfaction; see below and **Figure 3**).

**Figure 4:**
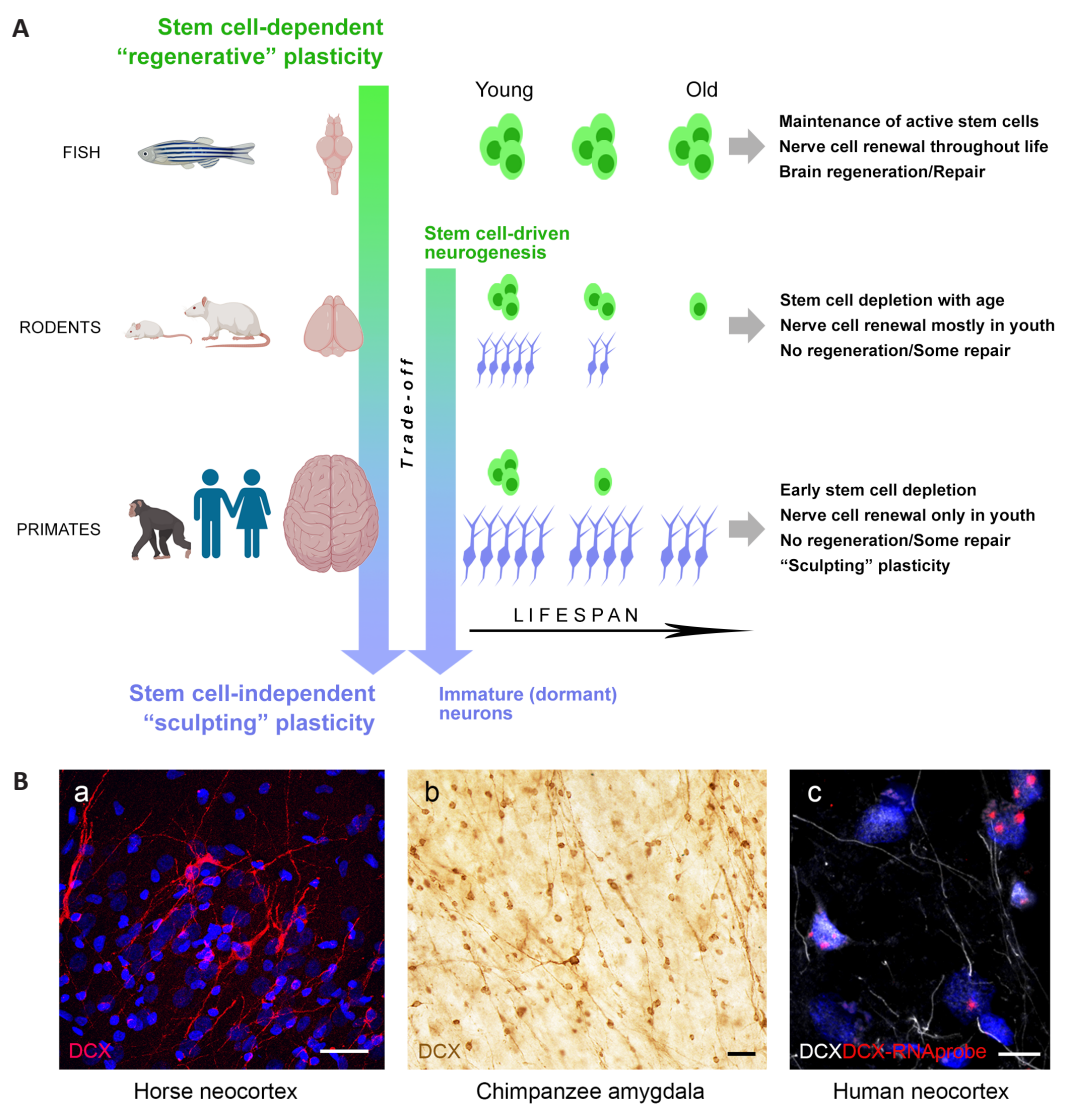
Different types of brain structural plasticity and their variation through phylogeny. (A) Stem cell–driven neurogenesis (including its age-related stem cell depletion/quiescence; green) versus stem cell–independent neurogenic plasticity (immature or “dormant” neurons; light blue) through phylogeny. In some non-mammalian vertebrates (e.g., fish), the neural stem cells and their niches are abundant and widespread in most areas of the nervous system, thus granting substantial neural cell renewal throughout life and processes of brain repair/regeneration after lesion (see Figure 2). In mammals, these are quite reduced in number and topographical location, with further differences between rodents and primates: their age-related depletion is far more evident (and early) in large-brained, gyrencephalic species. These species host larger numbers of cells in arrested maturation (immature neurons; light blue), widespread in the cerebral cortex and amygdala, where they provide a sort of “neurogenesis without division” (stem cell–independent). Overall, apart from a general reduction in the potential for structural plasticity through phylogeny, a shift occurs from “regenerative” forms of plasticity (requiring stem cell division and also granting continuous cell renewal) to forms of non-dividing, stem cell–independent neurogenesis linked to brain development, growth, and refinement (“sculpting” plasticity) rather than to brain repair. The size of cells (stem cells and immature neurons) indicates the different population sizes, while their number refers to maintenance throughout the lifespan. (B) Some examples of doublecortin-immunoreactive (DCX^+^) immature neurons in large-brained, gyrencephalic mammals. High numbers of DCX^+^ neurons can be found in the cortical layer II of adult horses and humans, as well as in the amygdala of chimpanzees (a, b, unpublished data; c, immunocytochemical staining in humans confirmed by RNAscope; reprinted with permission from Ghibaudi et al., 2023b). Scale bars: 50 µm in a and b; 10 µm in c. Created with BioRender.com.

In a phylogenetic profile, the complexity of evolutionary aspects hidden behind the phylogenetic reduction of neural regeneration suggests that a sort of trade-off between regeneration and plasticity has occurred, with the former decreasing with increasing brain complexity and the latter increasing with increasing brain complexity and lifespan (**Figure 4**). The reduction in regenerative plasticity with an increased capability to refine late-developing neurons/circuits is visible in a second type of trade-off that occurs between proliferative (stem cell–driven) and non-proliferative neurogenic mechanisms (**Figure 4** and section below).

## Case of Protracted Neurogenesis and the Trade-Off Hypothesis

A solid demonstration for the occurrence of adult neurogenesis in mammals was provided by studies performed in the nineties (Lois and Alvarez-Buylla, 1994; Kuhn et al., 1996) confirming previous findings that persistent cell division can give rise to new neurons at some locations of the mature brain (Altman and Das, 1965). The characterization of true stem-cell niches that enabled continuous neurogenesis (Doetsch et al., 1999; Gage, 2000) was then recalibrated by the observation of a progressive decrease in the neurogenic rate with time (Kuhn et al., 1996; Ben Abdallah et al., 2010; Charvet and Finlay, 2018; Snyder, 2019; Arellano and Rakic, 2024) due to age-related stem cell depletion and/or their entry into a state of quiescence (Encinas et al., 2011; Urbán et al., 2019; Blasco-Chamarro and Fariñas, 2023). Stem cell–driven neurogenesis in mammals is now accepted as a developmentally regulated process and mostly a juvenile feature that is further restricted to very early stages in humans (the postnatal periods for the lateral ventricle–olfactory bulb system; adolescence for the hippocampus; Sanai et al., 2011; Cipriani et al., 2018; Sorrells et al., 2018; Seki et al., 2019; Arellano and Rakic, 2024; Simard et al., 2024; Sorrells, 2024). For this reason, the notion of “adult” neurogenesis should be replaced by that of “protracted” neurogenesis (Bonfanti and Peretto, 2011; Snyder, 2019), which is a delayed developmental process needed to refine some brain circuits by taking advantage of experience (Kempermann, 2019; Semënov, 2019; Seki, 2020; Cushman et al., 2021). Especially in humans and other long-living mammals, neuronal plasticity can be the consequence of some developmental processes that remain active in the postnatal brain, thereby increasing the opportunity for consistent modification of the brain structure while the organism explores the world (Bonfanti and Peretto, 2011; Cushman et al., 2021; Sorrells, 2024).

Knowledge gathered in the last thirty years on this subject leads to some considerations that complicate the dream of regenerative medicine: (i) considering the brain anatomy, size, and energy costs, the concept of stem cell–driven neurogenesis does not fit well with the large, complex brains of long-living mammalian species (Paredes et al., 2016; Parolisi et al., 2018; Duque and Spector, 2019; Bonfanti et al., 2024); (ii) in all species, the juvenile neurogenic processes mostly operate to “sculpt” the vision of the world (based on the experiences of the animals that live in that world), rather than serving as a regenerative pool for brain repair; and (iii) large brains, endowed with higher cognitive functions and complex social interactions, are most common in long-living species that would take advantage of a delayed developmental “sculpting” plasticity (Kempermann, 2019; Semënov, 2019; Cushman et al., 2021), rather than brain repair (Weil et al., 2008). Overall, stem cell–dependent adult neurogenesis seems to fall into a typical example of a “narrowing road” in brain structural plasticity, linked to a progressively reduced availability of neural stem cells (number, distribution, and activity) through phylogeny (Encinas et al., 2011; Paredes et al., 2016; Cipriani et al., 2018; Parolisi et al., 2018; Sorrells et al., 2018; Bonfanti et al., 2024; **Figures 3** and **4**).

Conversely, recent studies have revealed a novel form of “neurogenesis without division,” consisting of neuronal populations that are generated prenatally (as most neurons of the brain are) but then enter a state of arrested maturation and remain immature for very long periods. They subsequently “awaken” (i.e., resume the maturational process) and complete their maturation and are finally integrated into the preexisting neural circuits (Gómez-Climent et al., 2008; Bonfanti and Nacher, 2012; König et al., 2016; Rotheneichner et al., 2018; Benedetti et al., 2020; La Rosa et al., 2020b; Bonfanti and Seki, 2021; Benedetti and Couillard-Despres, 2022; Alderman et al., 2024). These cells have been well characterized in the mouse piriform cortex layer II and, as mentioned above, are provisionally referred to as immature or dormant neurons (Gómez-Climent et al., 2008; Luzzati et al., 2009; Bonfanti and Nacher, 2012; König et al., 2016; Rotheneichner et al., 2018; Benedetti et al., 2020; Bonfanti and Seki, 2021; Benedetti and Couillard-Despres, 2022). While very little is currently known regarding the mechanisms that stop the maturation process or the mechanisms and stimuli that induce the awakening of the immature cells (although one possible signal for resuming maturation has been identified in PSA-NCAM depletion, and early life stress can alter maturation; Coviello et al., 2021; Abellán-Álvaro et al., 2024), their fate has been described in the piriform cortex using DCX-Cre-ERT2/Flox-EGFP transgenic mice (Rotheneichner et al., 2018; Benedetti et al., 2020, 2023). Following the steps involved in the entire maturation of the DCX/GFP^+^ cells, from small bipolar elements to large highly ramified pyramidal neurons (complex cells), enables visualization of the fully mature neurons (even after they have lost their markers of immaturity) as they become the glutamatergic principal neurons of the piriform cortex layer II (Rotheneichner et al., 2018; Benedetti et al., 2020). Although some maturing cortical immature neurons start to emit an axonal initial segment (Benedetti et al., 2020), how their axons can find the pathway to the final target in mature nervous tissue remains far from clear.

Two recent studies that analyzed the maturation time course of the cortical immature neurons in mice at different ages have shown a progressive age-related decrease resembling that described for adult neurogenesis (Ghibaudi et al., 2023a), while also noting that most of the neurons, including the small number that persist at very elderly stages, do awaken during the lifespan (Benedetti et al., 2023). Surprisingly, the immature neurons undergo remarkable phylogenetic variation during evolution. Whereas they are restricted to the paleocortex in rodents, they extend into the whole neocortex of large-brained gyrencephalic species and display far higher densities (one order of magnitude difference exists in the neocortex between mice and primates or carnivora; Palazzo et al., 2018; La Rosa et al., 2020a). Hence, these immature (dormant) cells provide new functional neurons at specific locations of the adult brain in a stem cell–independent manner, and form a large reservoir in gyrencephalic species, thus representing a first case of “enlarging the road” and expanding the substrate for neurogenic plasticity to higher-order brain regions, such as the cerebral cortex (Piumatti et al., 2018; La Rosa et al., 2020a; Bonfanti et al., 2024; **Figure 3**).

In the last few years, other reports have described very similar immature cells in the amygdala of different mammals (Fudge et al., 2012; Piumatti et al., 2018; Chareyron et al., 2021; Ghibaudi and Bonfanti, 2022; McHale-Matthews et al., 2023; Alderman et al., 2024; Ghibaudi et al., 2024), including humans (Martí-Mengual et al., 2013; Sorrells et al., 2019). Two of these reports used injections of the thymidine analog bromodeoxyuridine (BrdU) during embryonic development to demonstrate the prenatal origin of these cells in mice (Alderman et al., 2024) and in a gyrencephalic mammal (sheep; Piumatti et al., 2018). The available evidence indicates that only a few immature cells are detectable in mice (Alderman et al., 2024), whereas large numbers have been described in primates (Fudge et al., 2012; Chareyron et al., 2021; McHale-Matthews et al., 2023) and humans (Sorrells et al., 2019). A systematic comparative analysis like the one performed in the cortex (La Rosa et al., 2020a) has been recently carried out in the amygdala of widely different mammals, confirming the existence of a striking phylogenetic variation from rodents to primates (Ghibaudi et al., 2024). On this basis, evolutionary pressures associated with ecological niche or neurodevelopmental constraints have been suggested to lead to the selection of different types of plasticity in various mammalian species and brain regions, thereby achieving a trade-off between stem cell–driven neurogenesis and non-dividing immature neurons (Bonfanti et al., 2024; **Figure 4**).

This evolutionary trade-off refers to situations in which a compromise (due to anatomical limitations or to limited resources that must be allocated among competing demands) occurs between two or more traits that offer distinct benefits but cannot be fully optimized concurrently (Heldstab et al., 2022). Apart from the higher energy costs of maintaining a larger pool of stem cells for continuous neurogenesis (especially in large-sized brains), a trade-off in plasticity might have been influenced by a shift in the importance of different brain structures/functions, driven by evolutionary pressures. For example, the existence of a large gyrencephalic brain with a reduced olfactory bulb size (e.g., primates) *versus* a small lissencephalic brain endowed with a large olfactory bulb (e.g., rodents) could reflect functional adaptations of different brain structures involved in navigation, with rodents relying heavily on olfaction and larger mammals exploiting the computational capabilities of their expanded neocortical circuits (Aboitiz and Montiel, 2015; Englund and Krubitzer, 2022; **Figure 3**). According to this view, rodents rely mostly on olfactory and hippocampal neurogenic activity, whereas primates rely mostly on cortical immature neurons. Both types of structural plasticity (with or without cell division) add new neurons, though with different mechanisms (either stem cell–dependent or –independent) and in different brain regions (Bonfanti et al., 2024). In other words, old cell types and cell markers have been conserved throughout vertebrate evolution (Luzzati et al., 2009) by adapting to new brain regions and functions (Bonfanti et al., 2024).

## Developmental “Sculpting” Plasticity

Film director Franҫois Truffaut was reported to say that “a man establishes himself between 7 and 16 years of age, and then he will live by using what he has learnt in that period.” Of course, this is an oversimplification, but this idea is strongly supported by the environmental sculpting hypothesis for brain plasticity in juvenile life (as originally proposed for young/adult hippocampal neurogenesis; see Cushman et al., 2021). Brain structural plasticity, including synaptic remodeling (Ismail et al., 2017; Villanueva Espino et al., 2020), adult neurogenesis (Ben Abdallah et al., 2010; Sanai et al., 2011; Cushman et al., 2012), and immature neurons (Varea et al., 2009; Ghibaudi et al., 2023a; Benedetti et al., 2023), is established as occurring mostly during the juvenile periods of life to enable the construction and consolidation of experience-dependent brain connections. This “developmental” plasticity (here, intended to mean all structural changes occurring during the protracted postnatal development) is consistently present across species, including those having lost regenerative potential.

In fact, this plasticity is particularly evident in large-brained gyrencephalic species and acquires special importance in the human brain, contributing to the shift in brain weight from 250 grams at birth to 1300 grams at the end of somatic growth (DeFelipe, 2011). Indeed, the sculpting theory becomes more interesting when considering long-living species, such as primates and humans, which are characterized by delayed brain and body growth—features that enable a long-lasting, progressive “training to life” based on experience. Sculpting plasticity would permit the individual to gradually create a “vision of the world,” a process in which environmental experience, intended as a combination of multifactorial and multimodal (inanimate and social) stimulations, would markedly change the structure of the young brain and affect individual behavior in the subsequent lifespan (Rosenzweig and Bennett, 1996; Kempermann, 2019). As summarized by Kempermann (2019), “experience and behavioral activity leave structural traces that over time substantially contribute to defining our individuality and personality,” a process that takes only a few months in mice but at least 20 years in humans and allows humans to grasp the world as a complex system. In the past few years, this concept has acquired robust neurobiological evidence and can be formalized in the statement by the French philosopher Edgar Morin in his work on complex thought: “A well-done head is better than a well-filled one” (Morin, 2007).

On this basis, the prevalence of sculpting plasticity in large-brained mammals, particularly humans, may have importance beyond adaptation to a changing environment (this is the common explanation given for plasticity in the animal world). Indeed, it could largely determine the good or the bad responses of individuals and ultimately their future. However, as these theories have mostly been developed in the context of the expanding field of adult neurogenesis (especially hippocampal neurogenesis) and have used mice as leading animal models, this has introduced a bias linked to the more recent discovery that stem cell–driven neurogenesis in large-brained, long-living species is mostly restricted to very early developmental stages (Ben Abdallah et al., 2010; Encinas et al., 2011; Sanai et al., 2011; Cushman et al., 2012; Sorrells et al., 2018). The “environmental sculpting hypothesis” of adult neurogenesis has been proposed for hippocampal connectivity and its memory representations (Cushman et al., 2021); however, most examples of juvenile structural plasticity might be considered to have the same potential for sculpting neural circuits and functions to represent the world in which the adult individual must live and survive (McEwen, 2016; Semënov, 2019; Villanueva Espino et al., 2020; Cushman et al., 2021). This “prospective” function of structural plasticity clarifies its prevalence in postnatal/young stages and may also explain why it has expanded in large-brained mammals, using the stem cell–independent form of the dormant neurons to invade the cortical and subcortical areas involved in high computational/cognitive/emotional functions (Bonfanti et al., 2024). Recent developments in this direction also suggest a possible explanation for the discrepancy in finding DCX^+^ immature neurons in the adult human hippocampus in the absence of an active stem cell niche (Sorrells et al., 2018; Moreno-Jiménez et al., 2019). These cells might represent neurons in arrested maturation that were generated years before (e.g., during adolescence, when the stem cell niche was still active; Zhou et al., 2022; Bonfanti et al., 2024; Simard et al., 2024). Yet, further research is needed to verify their birth dates in gyrencephalic, long-living species.

The finding of large reservoirs of lifelong persisting immature neurons in the neocortex and amygdala may extend the experience-dependent sculpting to a more general property of brain function, as the animal species showing a preference for neurons in arrested maturation are those that show the characteristic of a complex brain capable of high cognitive functions (Palazzo et al., 2018; Sorrells et al., 2019; La Rosa et al., 2020a; Chareyron et al., 2021; La Rosa and Bonfanti, 2021; Page et al., 2022; Bonfanti et al., 2024), a characteristic finding its maximal expression in humans (Pascual-Leone et al., 2005; Han and Ma, 2015; Sherwood and Gómez-Robles, 2017; Lancaster, 2024). These facts indicate that the trade-off that occurred at the structural level between different types of plasticity (stem cells *versus* cells in arrested maturation) also involved a shift at the functional level, from a “navigating” role in the olfactory bulb and hippocampus (adult neurogenesis, mostly in rodents) to complex computational, emotional, and social roles in the neocortical and amygdala circuits (immature neurons in large-brained species; Bonfanti et al., 2024).

**Figure 5:**
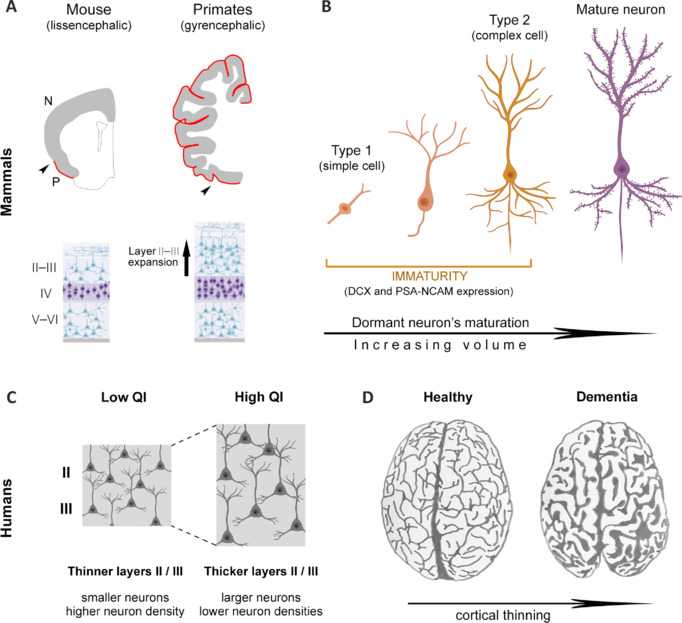
Possible contributions of immature neurons to cortical layer II/III thickness. (A) Across their progressive maturation through the lifespan (shifting from small type 1 cells to large, complex type 2 cells), the dormant or immature neurons of the cortical layer II increase their volume because of increased cell soma size and complexity of dendritic arborization. (B) Considering the high number of immature neurons present in the neocortex of gyrencephalic mammals (estimated to be around 2 million/hemisphere in chimpanzees, compared with only a few cells in mice), they may contribute to the different thicknesses of layers II/III described in these species. Red line: extension of the cortical layer II hosting immature neurons; arrowhead, limit between paleocortex (P) and neocortex (N). (C) In humans, interindividual variation of cortical layers II and III has been associated with different IQ scores, a higher IQ corresponding to increased neuropil volume and layer thickness. Even in pathological states, such as dementia (D), sulcal widening is a typical macroscopic sign of cortical mantle thinning. Overall, the existence of cortical immature neurons in the neocortex of large-brained mammals may be the structural substrate for a potential increase of neuropil volume and related connectivity. Figure assembled based on data from Rotheneichner et al., 2018; La Rosa et al., 2020a; Galakhova et al., 2022; de Sousa et al., 2023a. Created with BioRender.com.

Plasticity-related (postnatal) changes are known to play a role in sculpting the brain structure based on experience-dependent factors and can affect the volume and microstructure of the neuropil (Nithianantharajah and Hannan, 2006). In this context, a worthwhile point to mention is that cortical immature neurons are in layer II and upper layer III (Seki and Arai, 1991) of the cerebral cortex, where they are thought to play a role in plasticity by integrating corticocortical information and by participating in associative learning (La Rosa et al., 2020a). Comparative studies have shown that carnivores and primates (i.e., animal species that display high densities of immature neurons in their entire neocortex) are characterized by an expansion of the upper layers (II and III) when compared with rodents (de Sousa et al., 2023; **Figure 5A**). The progressive maturation of the immature neurons occurs continuously in those layers (Rotheneichner et al., 2018; Benedetti et al., 2020), thereby producing an increased volume that is linked to the shift from type 1 cells (small soma, simple morphology) to type 2 cells (larger and complex neurons; Piumatti et al., 2018; La Rosa et al., 2020a; Ghibaudi et al., 2023a; **Figure 5B**) and possibly contributing to the layer thickness. Hence, imagining that the progressive sculpting of the cortical immature neuron cell population across a lifetime would affect cortical thickness is not unreasonable. For instance, a study carried out on the human cortex middle temporal gyrus demonstrated an association between cortical thickness and intelligence quotient (IQ) scores in healthy individuals, particularly those showing differences in the thicknesses of layers II and III (Choi et al., 2008; Heyer et al., 2022). Layers II–III were thicker and had lower neuron densities and larger neurons in the subjects with higher IQ scores than in those with lower IQ scores (Heyer et al., 2022). The pyramidal neurons also had larger and more complex dendritic trees in layers II–III in subjects with higher IQ scores, thus having more dendritic spines and more physical room to receive synaptic inputs (Galakhova et al., 2022; **Figure 5C**).

## Role of Brain Plasticity in Large-Brained Mammals and Its Translational Zest

The above sections support the idea that humans, or more generally primates and other gyrencephalic species, would have developed stem cell–independent sculpting plasticity, rather than regenerative strategies, across phylogeny. This makes sense if plasticity is considered to represent a transversal tool used by different brains to perform functions that can differ depending on the ecological niche of each animal species (Barker et al., 2011). Nevertheless, a large amount of research in neuroscience and the field of neural plasticity is driven by the goal of fostering brain repair, and most of the funding granted by charities or big pharmaceutical companies goes in that direction. This is perhaps the reason why most scientists studying plasticity are primarily interested in finding mechanisms of/effects on brain repair and endeavoring to associate plastic changes with a translational outcome (Martino et al., 2011; Bao and Song, 2018; Seblani et al., 2024). This is surely an important goal to be pursued despite obvious difficulties (Weil et al., 2008; Bonfanti, 2016), although present evidence now clearly indicates that the potential for structural remodeling provided by brain plasticity can serve two main purposes: i) the refinement of neural networks in the healthy brain to build up a young individual’s vision of the world and to adapt an adult to physiological or environmental changes (this seems to be a transversal and primary role) and ii) compensation for the loss of structure/function as a consequence of lesion, injury, or pathological states, producing cell/tissue repair only in some cases (which can be considered a byproduct of evolution). Across phylogeny, these roles are mixed differently, with a prevalence of cell renewal/regeneration in simple brains and of sculpting non-regenerative plasticity in mammals (as summarized in **Figure 4**).

Recent developments introducing the concept of dormant neurons, along with the discovery of their prevalence in large-brained species, seem to confirm that forms of stem cell–independent structural plasticity (not involving cell division) would be a prevalent choice among long-living mammals endowed with complex brains and high cognitive/computational capabilities. As stated by Bullock (2007), two important factors to consider in the evolution of complex nervous systems are (1) how we define and measure complexity and (2) which level of organization is of interest (e.g., from the single neuron or synapse to social behavior; Krubitzer, 2009). In this review, we considered the phylogenetic variation in the occurrence, location, and amount of structural plasticity as a further level of complexity, and we underlined the view that the “enlarging road,” consisting of an increase of immature neurons from mice to primates, seems to favor processes of sculpting plasticity rather than regenerative strategies (**Figures 3**–**5**).

**Figure 6:**
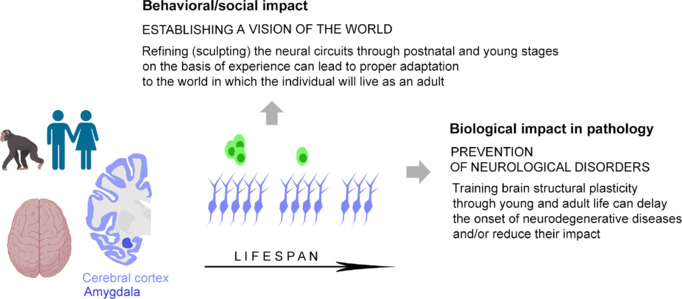
Possible dual impact of “sculpting” plasticity in large-brained mammals. The abundance of immature neurons in high-order processing brain regions, such as the cerebral cortex and the amygdala, of large-brained, gyrencephalic mammals can play a dual role at young and old ages (left and right part of the arrow, respectively): (i) as a prevalent substrate for developmental, “sculpting” plasticity (along with neurogenesis still present during youth; green cells), which allows the growing brain to refine its circuits on the basis of experience while building up a “vision of the world” (a process delayed in time in humans and primates, potentially having a remarkable impact on the social life of adult individuals), and (ii) as a form of prevention (brain or cognitive reserve) of age-related cognitive impairments or incoming neurological disorders. In an evolutionary context, the reduction in stem cell–driven neurogenesis (green) and the increase in non-dividing immature neurons (for the trade-off hypothesis, see Bonfanti et al., 2024 and Figure 4) has led large-brained mammals (mostly long-living) to invest more in a slow and progressive sculpting of the young brain, at the expense of the regenerative capacity. Apparently, this shift was advantageous to the survival of the species and (especially in humans) their capabilities to master the world. Created with BioRender.com.

Although experimental studies are still lacking regarding the possible modulation of immature neurons after lesion or environmental changes, we know that the cortical immature neurons are in place in their final location within cortical layer II since their birth during embryonic neurogenesis and are already specified to become glutamatergic principal neurons in that same layer (Gómez-Climent et al., 2008; Rotheneichner et al., 2018; Benedetti et al., 2020, 2023; Benedetti and Couillard-Despres, 2022). These features strongly suggest that the immature cells play a primary role in cortical plasticity by progressively integrating into the pre-existing circuits in which they were waiting in standby (Rotheneichner et al., 2018; Benedetti et al., 2020, 2023; Benedetti and Couillard-Despres, 2022), rather than being mobilized for brain repair. In this view, recruiting the neurons held in arrested maturation through life would contribute to the refinement of the cortical circuits (a similar process likely occurs in the amygdala; Piumatti et al., 2018; Sorrells et al., 2019; Page et al., 2022; Alderman et al., 2024; Ghibaudi et al., 2024), thus contributing to sculpting the vision of the world in which the animal actually lives during its youth and will live in as an adult, in a similar manner to what has been proposed for the stem cell–driven adult neurogenesis occurring during juvenile ages (Sailor et al., 2017; Kempermann, 2019; Semënov, 2019; Cushman et al., 2021; **Figure 6**).

Another important (and often neglected) principle of brain plasticity considered under a comparative approach is that regional specializations can indicate functional adaptations (Barker et al., 2011). Thus, the shift from neurogenic niches in the olfactory system, hippocampus, and hypothalamus to the cerebral cortex and amygdala may represent a shift from more instinctive functions to high/complex (computational and social) cognitive capabilities (see Aboitiz and Montiel, 2015; Bonfanti et al., 2024). The current view is that evolutionary events occurring in apes and humans provided thicker cortical gray matter and enabled more extensive cortical connectivity, especially through elaboration of superficial intracortical connections (Lancaster, 2024). Instead of increasing the number and diversity of neurons within cortical modules, the number of modules increased, with increases in the number and diversity of cortical fields and the development of much more elaborate dendritic branching to allow more elaborate processing capability (Galakhova et al., 2022; Lancaster, 2024). This is why the cerebral cortices of gyrencephalic species can be considered “more complex” than those of rodents, despite their substantially similar histology and cytoarchitecture (Krubitzer, 2009; Sherwood and Gómez-Robles, 2017; Lancaster, 2024). Accordingly, immature neurons are placed in the neocortices of gyrencephalic species, thereby increasing the volume of their dendritic branching during maturation and potentially increasing the cortical neuropil volume (Benedetti and Couillard-Despres, 2022; **Figure 5B**). In the above section, we discussed how this type of thickness modification might be a consequence of experience-induced sculpting. Even in cases of cognitive decline, both in healthy aging and pathological brains (e.g., dementia), a strong correlation with morphological changes, referred to as cerebral atrophy, is observed. These changes can include neurodegeneration, cortical thinning, sulcal widening, hippocampal atrophy, white and gray matter volume loss, ventricular enlargement, and loss of gyrification (Pini et al., 2016; Tang et al., 2024; **Figure 5D**). This knowledge, apart from explaining how the brain can maintain its efficiency (and cortical thickness; Narr et al., 2007; Choi et al., 2008; Heyer et al., 2022), may have important implications for preventing cortical thinning and cognitive decline (**Figure 6**). For instance, recent studies suggest that plasticity across life, and particularly the summation of progressive changes during juvenile life, may increase or decrease the risk of developing a psychiatric disorder (Paus et al., 2008; Forrest et al., 2018; Patel et al., 2021).

Another emerging role of brain plasticity is linked to the idea of brain reserve (or cognitive reserve or brain maintenance)—a concept used in neurology to explain discrepancies between the individual level of brain pathology and the expected cognitive performance (Scarmeas and Stern, 2003; Barulli and Stern, 2013; Stern et al., 2018). The concept is based on neuroimaging or anatomical measures (e.g., brain volume, cortical thickness, synaptic count, dendritic branching) carried out in postmortem human brain tissues, with the plastic changes being the result of (or the biological substrate for) structural modifications across the lifespan. Despite the obvious difficulties encountered in studying human brains, a clearly emerging development is that some individuals with Alzheimer’s disease-like neuropathology do not become symptomatic during their lifetime, and that individuals with greater brain reserve may require more pathology to manifest clinical dementia (Erten-Lyons et al., 2009; Kramer et al., 2011; Zolochevska and Taglialatela, 2016; Darwish et al., 2018; Giovacchini et al., 2019). Patients who remain cognitively intact despite having an accumulation of amyloid plaques and neurofibrillary tangles like those observed in fully symptomatic Alzheimer’s disease (AD) are defined as having “non-demented AD,” “cognitively successful aging,” “asymptomatic AD,” or “resilient AD” (Bjorklund et al., 2012; Zolochevska and Taglialatela, 2016; Giovacchini et al., 2019). Protective/compensatory mechanisms would be responsible for resistance to neurological diseases or significant delays in the emergence of clinical symptoms (**Figure 6**). By virtue of the plastic changes induced and strengthened by lifestyle throughout early and young ages, the aging brain would be able to contend with degeneration and damage by adopting pre-existing cognitive processes or recruiting compensatory processes (Scarmeas and Stern, 2003; Nithianantharajah and Hannan, 2006; Erten-Lyons et al., 2009; Cabeza et al., 2018; Navakkode and Kennedy, 2024).

Different lifetime experiences, such as educational/occupational attainment and leisure activity, will be tools for prevention that can be imagined, evolutionarily speaking, as a sort of compensation for the loss of regenerative capabilities (Snowdon et al., 1996; Scarmeas and Stern, 2003; Darwish et al., 2018). In other words, the brain reserve could be imagined as a mixture of good brain maintenance and cognitive reserves, with the former considered as the hardware (all processes underlying the structural preservation of the brain with age) and the latter as the software (brain functioning explained by factors beyond mere brain structure; Habeck et al., 2016). Accordingly, the cognitive reserve should not be viewed as a single underlying process; rather, it should be considered a set of different processes acting at different temporal and spatial scales, among which the widely expanded reservoir of immature neurons present in large-brained mammals might represent an important substrate (La Rosa et al., 2019). Again, phylogenetic variation plays a role across mammalian species, since recent reports on the occurrence of cortical immature neurons at different ages indicate an age-related progressive reduction in rodents (Varea et al., 2009; Ghibaudi et al., 2023a) but substantial maintenance in large-brained, long-living species (La Rosa et al., 2020a; Ghibaudi et al., 2023a), including humans (Coviello et al., 2022; Li et al., 2023; see **Figure 1**, bottom). Hence, the dormant neurons, having undergone a substantial enlargement in their numbers, as well as changes in anatomical location and persistence in age across phylogeny, can represent a potential reservoir of young neural elements in gyrencephalic, long-living mammals.

In conclusion, the topic of immature neurons is only in its infancy, and further studies are needed to confirm the current hypotheses and unravel the physiological role these cells play in determining brain plasticity. The first real discovery was the finding that an important form of structural (neurogenic) plasticity has shifted during evolution from stem cells, the olfactory bulb, and the hippocampus to non-dividing neurons, the cerebral cortex, and the amygdala following the increase in brain size and gyrification. The good news, which should prompt further research of this type, is the now-established fact that dormant neurons are part of an enlarged road in the phylogenetic trend of brain structural plasticity for gyrencephalic mammals and thus represent a translational zest for humans, as an opportunity for preventive approaches in neurology.

**Additional file:** *Open peer review report 1.*

OPEN PEER REVIEW REPORT 1

## Data Availability

*Not applicable*.
