# Supplementary material for: Brain structural plasticity in large-brained mammals: Not only narrowing roads
Source: Neural Regen Res. 2025 Mar 25;21(5):1669–80. doi: 10.4103/NRR.NRR-D-24-01438 (PMC12694648; doi:10.4103/NRR.NRR-D-24-01438)
Supplement: Supplementary file 1 [file NRR-21-1669_Suppl1.pdf]

# OPEN PEER REVIEW REPORT 1

**Name of journal:** Neural Regeneration Research
**Manuscript NO:** NRR-D-24-01438
**Title:** Brain structural plasticity in large-brained mammals: not only narrowing roads
**Reviewer's Name: Juan Nacher**
**Reviewer's country: SPAIN**

## COMMENTS TO AUTHORS

Overall evaluation on article quality:
This is a very interesting and necessary review, which recapitulates our knowledge on the structural plasticity of the adult brain and particularly the role that immature/dormant neurons play in this process. The review is very well written, and the figures are very explicative and of good quality. I only have some minor suggestions that I think can improve the manuscript.

1. I think that the author should mention also the presence of adult neurogenesis and regeneration in certain reptiles, as well as the presence of immature neurons (which his laboratory and others described many years ago).
2. It could be also interesting that in lizards there is evidence of an interaction between microglial cells and adult neurogenesis/regeneration (Lopez-Garcia et al., 1994).
3. In page 8 line 43 the author states that postnatal developmental changes were the primary goal of introducing neuronal plasticity in organisms, while brain repair was a byproduct of evolution. I think it should be also considered that neuronal plasticity can be also the consequence of some developmental processes still active in the postnatal brain.
4. There is an aspect that the author does not mention and I think it is a very important point, which has received little attention: How the immature/dormant neurons achieve their development, including neurite outgrowth and synaptogenesis, in an environment that is highly restrictive for these proceses.
5. Page 9/35 I think that it should be mentioned that some studies claim that adult hippocampal neurogenesis still exists in humans. While this is a very controversial subject, I think the studies should be mentioned. In fact they are asl interesting because they also suggest the presence of dormant/immature neurons in the adult hippocampus.
6. In page 9 line 27 it would be interesting to mention that adult neurogenesis in aged rodents can be modulated by adrenalectomy and NMDA receptor antagonists.
7. The author claims that nothing is known about the process awakening these cells, but there are some studies that indicate that the polysialylated form of NCAM, different monoamines and NMDA receptors participate in this process.
8. Page 10 line 44. The time course of age-related reduction in immature/dormant neurons in the piriform cortex was first described in rats (Varea et al., 2009).
9. I think that it also could be interesting to mention that the population of immature/dormant neurons of cortical layer II is affected in some animal models of neurological and psychiatric disorders or under the effects of stress or stress-related hormones.
10. In page 17, line 6, I think it is necessary also to mention tha article by Coviello et al.(2022), which described the phenotype and distribution of dormant neurons in the human cerebral cortex.

Timeliness evaluation on article:
This is a timely review in the actual context of the study of neural plasticity from different perspectives. The role of dormant neurons in this plasticity has not been sufficiently explored and the author does an excellent job in reviewing the

Scope evaluation on article:
The review article has a comprehensive scope, covering a broad range of relevant studies and providing

a thorough overview of the topic.

Direction evaluation on article:
The review article has a clear and well-defined focus, presenting the main research questions or objectives that guide the review process.

Novelty evaluation on article:
Score 3: The review article presents a highly novel and insightful synthesis of existing literature, offering new perspectives or frameworks that enrich the understanding of the field.
